# Study on discriminant method of rock type for porous carbonate reservoirs based on Bayesian theory

**DOI:** 10.1038/s41598-021-98154-x

**Published:** 2021-09-20

**Authors:** Xinxin Fang, Hong Feng

**Affiliations:** 1 China Coal Research Institute, Beijing, 100013 China; 2 China Coal Technology and Engineering Group Xian Institute, Xi’an, 710077 Shaanxi China

**Keywords:** Solid Earth sciences, Energy science and technology

## Abstract

Rock typing is an extremely critical step in the estimation of carbonate reservoir quality and reserves in the Middle East. In order to recognize the rock types of carbonate reservoirs in the Mishrif Formation better, classify the reservoirs accurately, and establish the permeability model in line with the study area precisely, it is necessary to study the recognition method conforming to the actual situation of the study area. The practice shows that the current recognition methods based on capillary pressure curve, flow unit and NMR logging data can effectively distinguish rock types, but a large number of accurate experimental data are required, which can only be applied in a few cored well, however, cannot be applied in the whole oil field. In this study, based on core, thin section, logging data, the sedimentary characteristics of carbonate reservoir, logging response of four rock types as well as porosity and permeability characteristics of Mishrif Formation in W are comprehensively studied. Based on Bayesian stepwise discriminant theory in multivariate statistics, the Bayesian discrimination model based on conventional logging data is established. The examining results showed that, compared with the description of logging and coring, the accuracy of Bayesian discriminant model and cross confirmation rate have achieved more than 80% for the original sample. Reliability verification showed that the matching degree of the rock type recognized in the non-cored well with the core and mud logging was as high as 90%, which matched the depositional environment of the entire region. The study results confirm the validity and generalizability of the Bayesian method to identify and predict rock types, which can be applied to the entire Middle East region to solve the problem of the lack of core data to accurately evaluate the quality of non-cored wells and accurately predict production, meeting the needs of actual reservoir evaluation and production development in the Middle East.

## Introduction

The Dunham rock typing method^1^ based on the support and content of mud and grain is currently adopted in the Middle East, Iraq, which divides carbonate rocks into mudstone, wackstone, packstone, grainstone^1–3^. In the comprehensive logging evaluation of carbonate reservoirs in the Middle East, accurate recognition of rock types is an extremely critical step for establishing an effective permeability interpretation model, accurate reservoir classification, and assessment of geological reserves. Due to the complex pore types, pore structures and rock fabric of the Mishrif Formation carbonate reservoir in the W oilfield, the heterogeneity is very strong, in addition the lack of data from the cored wells in the Middle East, which increase the difficulty of recognizing rock types in this area. Although it is an effective geological method to recognize rock types by relying on core, casting thin section and logging, it depends greatly on core, which leads to heavy workload and high cost^4–6^. Therefore, recognition methods based on logging data is still the main choice. Though, electrical imaging logging data can be used to recognize rock types efficiently, it is rare. It is necessary to study a method of rock type recognition based on conventional logging data to predict rock type continuously for most non-cored wells. The Mishrif Formation carbonate reservoirs in the Middle East are very similar in sedimentary period, sedimentary and diagenetic environment. A certain type of strata in the same sedimentary environment has a set of specific logging parameter values (including logging response values and the extraction of information related to rock types from logging data)^7–10^. Hence, the method of predicting reservoir rock types in the Mishrif Formation based on conventional logging data can be widely used in the Middle East.

Presently, little research on the method of recognizing the rock type of porous carbonate reservoir based on conventional logging data has been done. In the actual work, the main methods are as follows. (1) The cross-plot method, which is based on different logging curves to distinguish the rock types in the plane intersection graph^11–14^. (2) The method based on the capillary pressure curves. Fournier clustered the capillary pressure curves with mercury intrusion experimental data, and compared the rock type with the curve types to automatically recognize rock types. (3) The method based on NMR logging data, experiments show that NMR T2 distribution spectrum of different types of rocks are different, Frank et al. classified the rocks in core wells into different types according to this feature in the process of studying the permeability model of carbonate reservoirs^15^. (4) Recognition method based on flow units. This method is based on the difference in porosity and permeability of different types of rocks. Leverett (1941) studied the microscopic pore structure and petroelectric characteristics of different carbonate reservoirs, and introduced the concept of J function to quantitatively characterize this difference, which was proportional to K/$\Phi$^16,17^.

These methods have their own characteristics in the recognition of different rock types. However, the cross-plot method is effective in recognition of rocks with single lithology, uncomplicated pore structure and uniform pore distribution, but not in the porous carbonate reservoirs of the Middle East. The latter three methods require a large amount of coring data and nuclear magnetic resonance logging data, which add extra cost in actual oil field production. In view of this, this paper introduces the Bayesian discriminant theory in multivariate statistics. Bayesian discriminant analysis is a statistical analysis method combined with effective selection of parameter and quantitative identification, and makes full use of sample information and prior information of parameters, when estimating parameters, Bayesian estimators usually have smaller variance or square error and can get more accurate prediction results. It could quantitatively evaluate the results of judgments made in hypothesis testing or estimation problems, rather than simple judgments of acceptance or rejection in frequency statistics theory. By comparing the posterior probability of all kinds of samples in this method, the classification of samples is determined, which has high accuracy and good stability, but impacted by sample numbers^18,19^. Based on 90 sample data of 12 cored wells, the sensitive logging parameters of different rock types are selected, then the Bayesian discriminant model of rock types is established, which can make full use of the prior information of logging parameters reflecting rock types, with smaller variance or square error in parameter estimation. It could achieve efficient identification of rock types, thereby reducing the degree of dependence on new logging methods and mercury intrusion data.

## Geological setting

**Figure 1:**
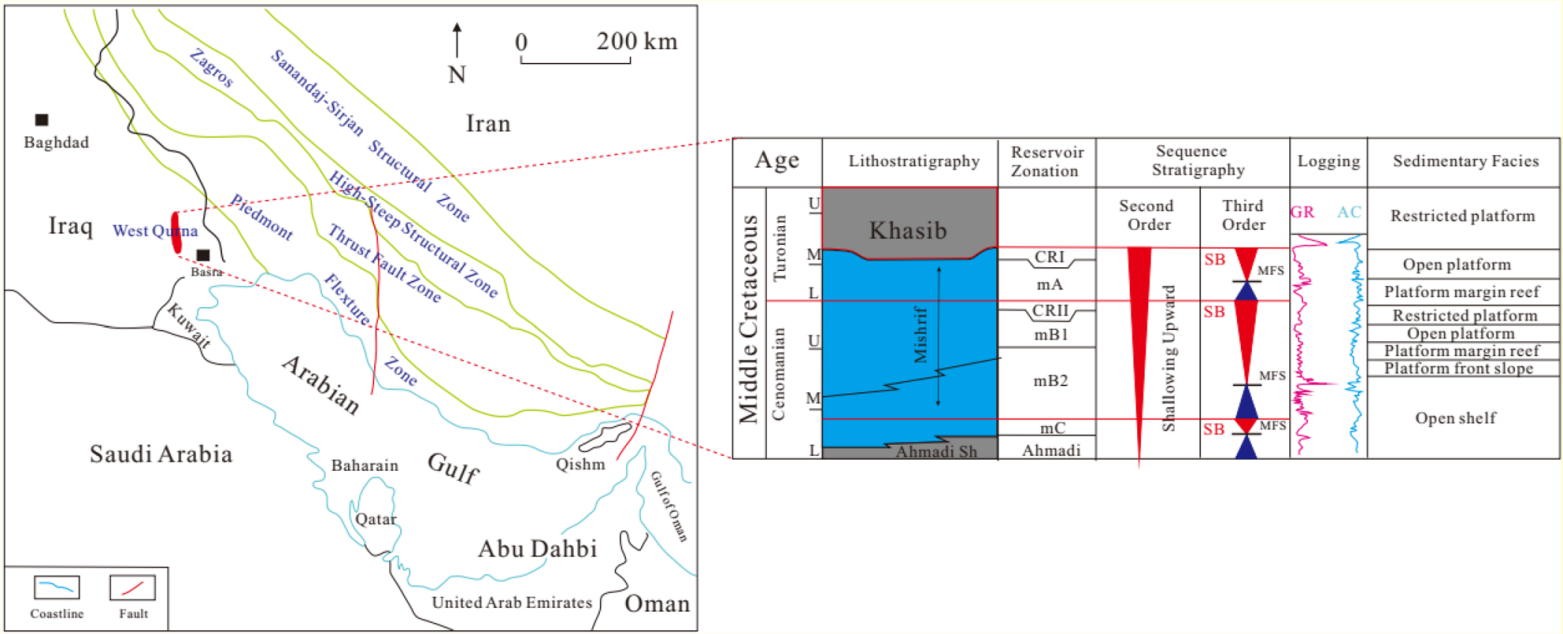
Regional tectonic location of study area, sequence and sedimentary characteristics of Mishirif Formation in the study area.

The Arabian Gulf is a shallow continental marginal sea located in a foreland basin that separates the Arabian shield in the west from the active fold-thrust belt of the Zagros Mountains in Iran^20–22^. W (referred as West Quarna) Oil Field is located in the southeast of Iraq, 50 km northwest of Basra City, tectonically belonging to the Arabian Plate, Dibdiba Depression, and the northward extension of Rumaila Anticline^23–25^. This block is about 50 km long and 14 km wide (Fig. 1). The Mishrif Formation in W Oilfield is a very important set of sequences in Mesopotamia Basin and widely developed in the Arabian Gulf. The reservoir was deposited on the passive margin throughout the Mesozoic, which was located in the eastern Arabian craton and was extensively covered by shallow sea^26,27^. The Mishrif Formation is the main oil reservoir in southern and central Iraq, which belongs to the shallow continuous carbonate deposits. The periodic changes of sea level resulted in the deep-water deposits, during which the outer continental shelf and basin deposits outside the Rumaila Formation was formed^28,29^. The formation of carbonate reservoirs in the Mishrif Formation is closely related to the tectonic evolution background of the Arabian Gulf Basin. At the beginning of the Precambrian, the Arabian plate proliferated and developed the basement of the Arabian Gulf basin. The late Precambrian-Middle Permian was the period of intracratonic sedimentary development. The Paleo-Tethys Ocean closed at the Late Permian, then the Arabian Gulf evolved as a passive continental margin on the southern margin of the New Tethys Ocean. During the Miocene, the New Tethys Ocean was closed and controlled by the Zagros orogeny, the Arabian Gulf area turned into an active tectonic plate margin, which continued to now^30,31^.

The Mishrif reservoir can be divided into one secondary cycle and three third cycles^8,32,33^. From top to bottom, it is divided into six layers: CRI, CRII, mA, mB1, mB2 and mC. The sedimentary facies can be divided into six sub facies: limited platform, open platform, platform margin reef, platform front slope and open shelf.

According to the drilling and logging data of 8 wells, the average thickness of carbonate reservoir in the Mishrif Formation in the study area is 302 m, and the proportion of the formation of different rock types in the total thickness is shown in Table 1. It can be seen from Table 1 that the largest thickness in the study area is packstone, accounting for 48.34% of the total thickness, followed by grainstone, third wackstone, and the thinnest is mudstone. It can be seen that grainstone, packstone and mudstone are the three main rock types of the Mishrif Formation (Fig. 1).

**Table 1:** Statistics of rock types of Mishrif Formation.

Rock type	Thickness/m	Proportion/%
Grainstone	137	45.36
Packstone	146	48.34
Wackstone	14	4.63
Mudstone	5	1.66

## Logging response, rock fabric and pore structure characteristics of different rock types

**Figure 2:**
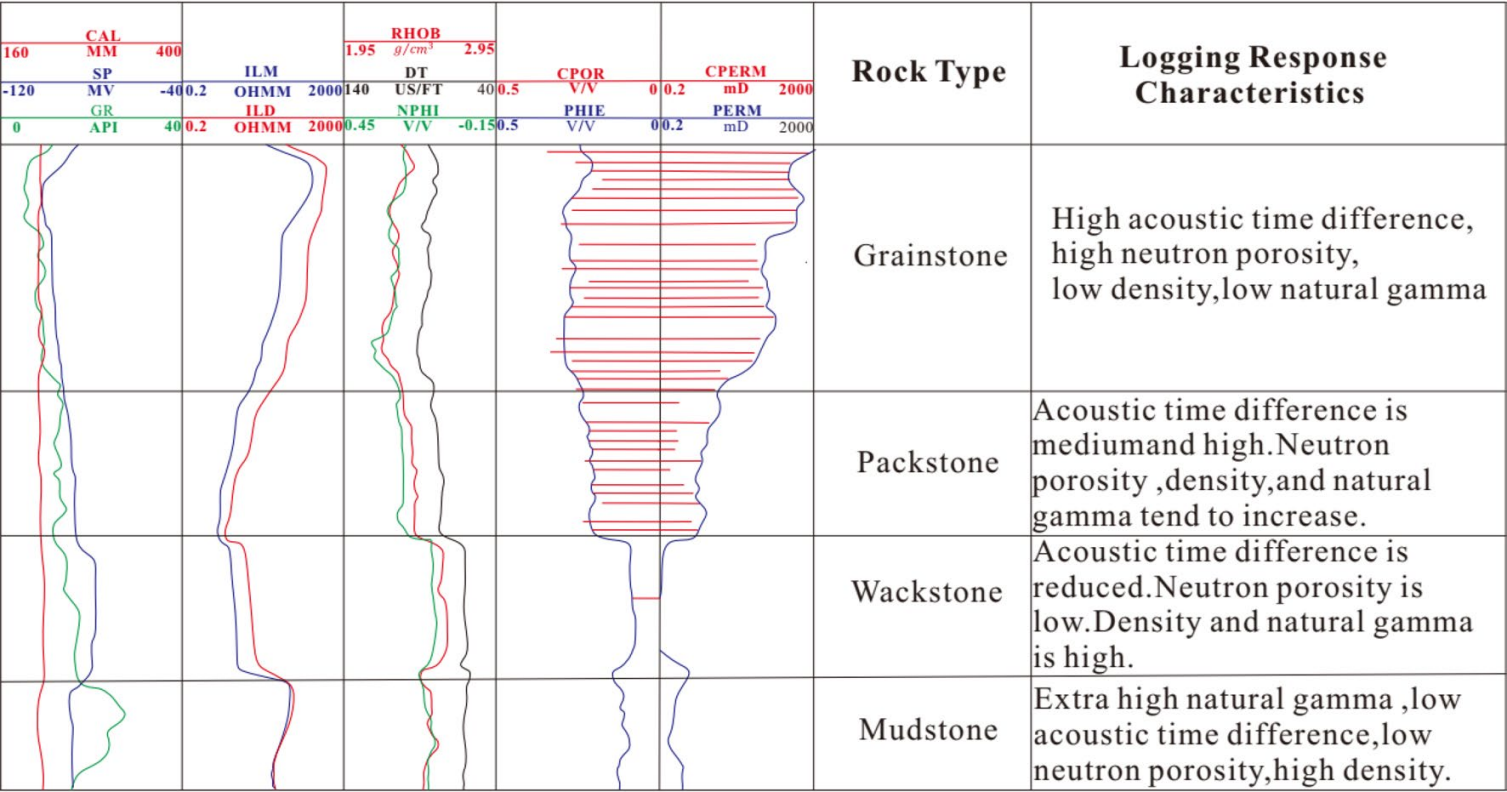
Logging response characteristics of different rock types.

The logging response characteristics of rock are the comprehensive reflection of rock fabric, structure, pore type and oil and gas bearing property. Taking well W-2 as an example, the logging response characteristics of four rock types, i.e., grainstone, packstone, wackstone and mudstone, were summarized based on core and logging data (Fig. 2).

**Figure 3:**
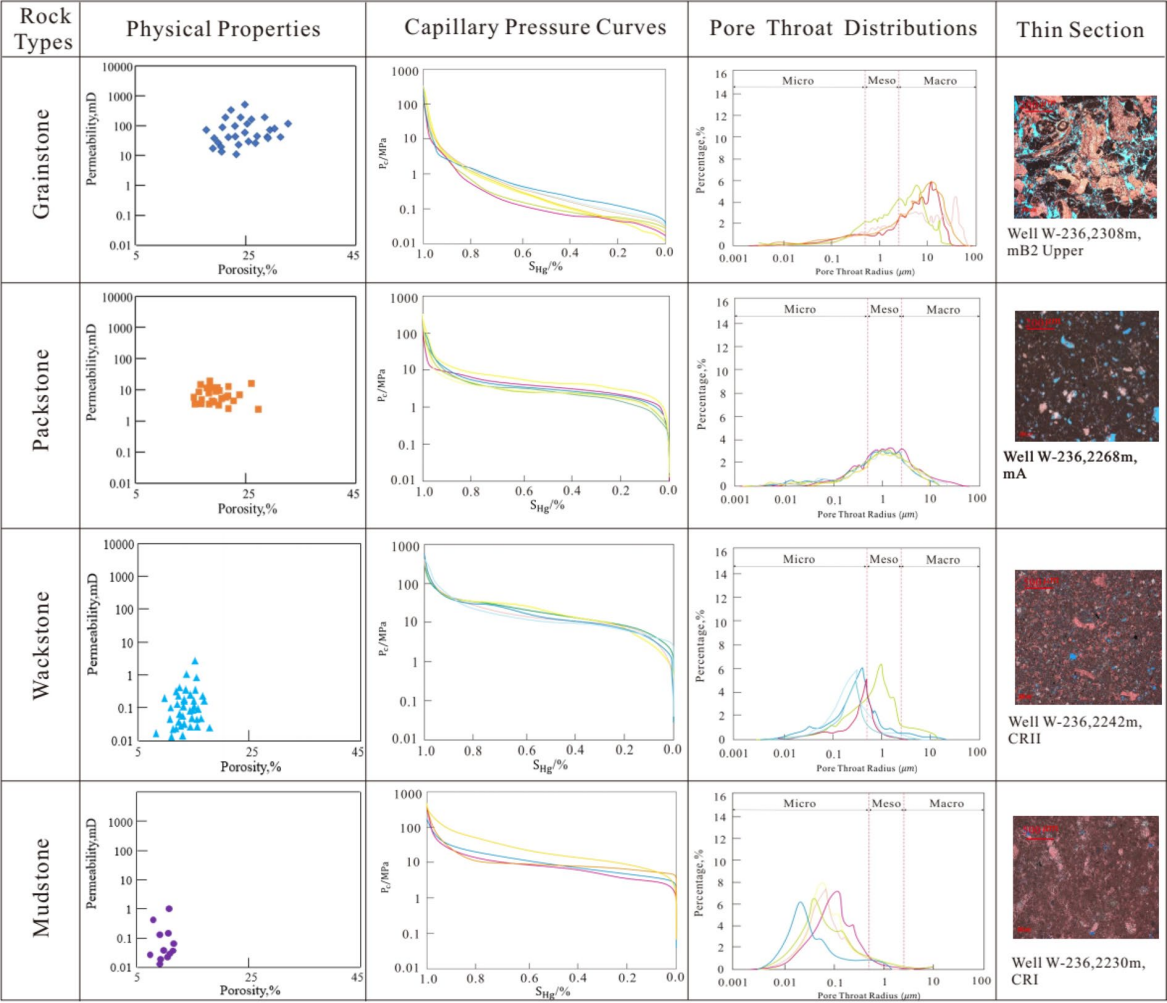
Relationship diagram of pore-throat radius, thin section and pore-permeability of different rock types.

Grainstone is mainly formed in high energy environment, with strong scouring effect by water, low mud content. Pore types are dominated by interparticle, moldic pore, and vugs, with large throat radius greater than 10 μm (Fig. 3). Natural gamma value is generally less than 8API.The acoustic time difference is obviously larger than 80 μs/ft. The neutron porosity is larger than 0.2 (Fig. 2). The density is less than 2.36 kg/m^3^. The reservoir quality is good, recognized as high porosity and permeability ones.

Packstone is developed in medium and high energy environment. When water velocity decreases, fine-grained sediment will be deposited. Compared with grainstone, natural gamma value has a tend to increase. Micropores, moldic, intergranular and intragranular pores are mainly found there. Due to impact of mud, primary porosity decreases accordingly, and acoustic time difference tends to decrease in the range of 74 to 80 μs/f, neutron porosity decreases in the range of 0.17–0.22, and density increases in the range of 2.36 to 2.52 g/cm^3^ (Fig. 2). The throat becomes thinner, pore throat radius varies from 1 to 10 μm (Fig. 3), permeability is weakened, and physical relationship is characterized by high porosity and low permeability.

Wackstone is formed in deep water with low water velocity and hydrodynamics, which makes it conductive for mud to deposit, resulting in natural gamma value to increase obviously. The primary pores are not developed and are dominated by micropores and moldic pores, with small pore throat radius in the range of 0.1–1 μm (Fig. 3). The acoustic time difference and neutron porosity are greatly reduced, resulting in acoustic time difference less than 62 μs/f and neutron porosity to vary from 0.08 to 0.15. Density is larger than 2.54 g/cm^3^ (Fig. 2). It is characterized as low porosity and permeability ones.

Mudstone is formed in a deep-sea environment, below the wave base, where water is quiet. The mud is accumulated in a large area with no effective pores and poor permeability, which is generally recognized as non-reservoir.

The four rock types have obvious differences in physical properties, pore structure, rock fabric, and logging response characteristics. Therefore, Bayesian discrimination model of rock types can be established based on this.

## Quantitative recognition of rock types based on logging

**Figure 4:**
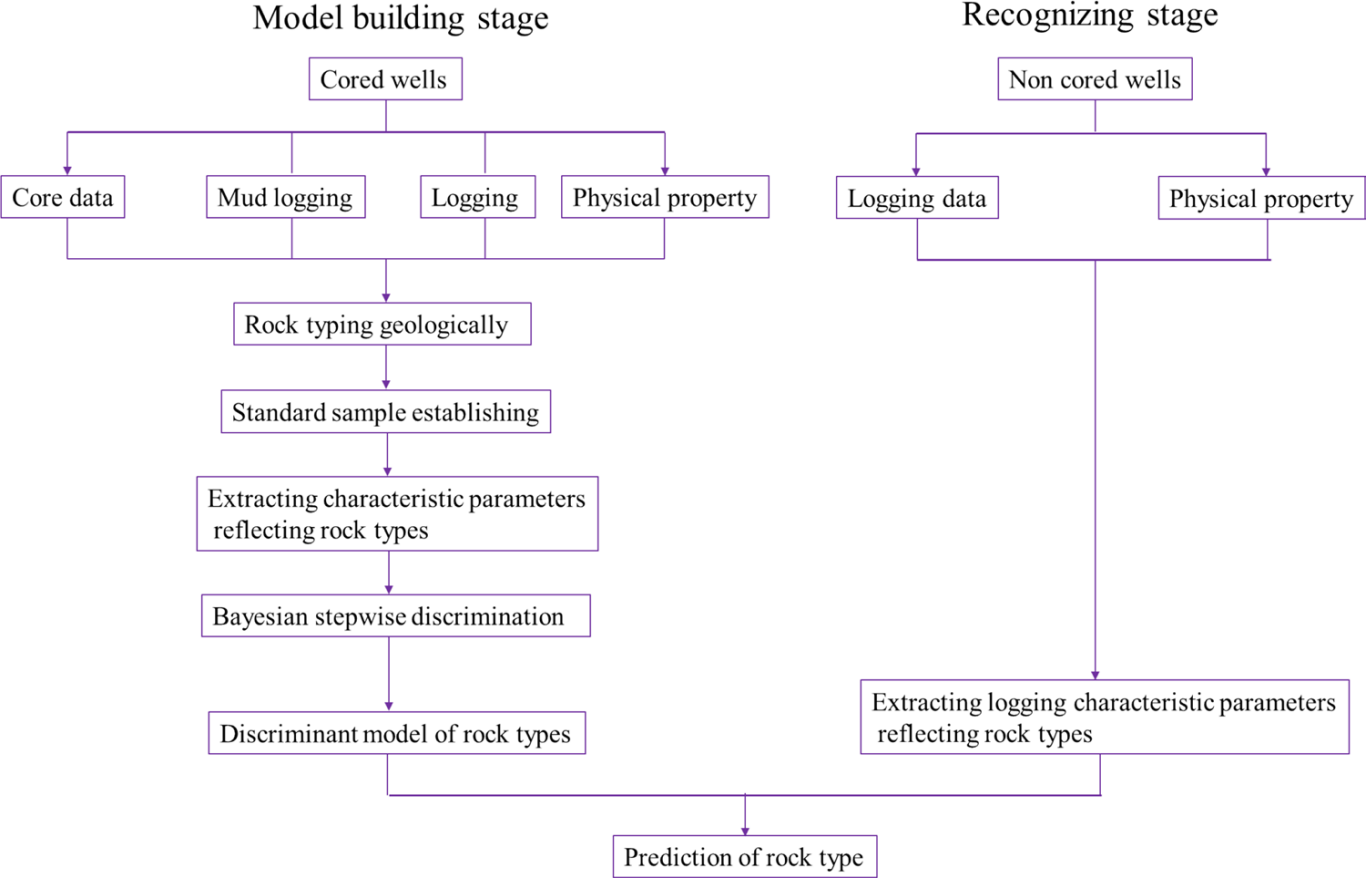
The process chart of Bayesian discriminant method in recognizing rock types.

Due to formation heterogeneity, complex pore structure, diverse pore types, and interference factors such as formation compaction and logging tools, single logging curve cannot accurately determine rock types. To solve this problem, after making full use of all logging curves in the study area, selecting sensitive logging parameters, quantitative discriminant model based on the Bayesian theory was established^34,35^ (Fig. 4). The logging response characteristics of rocks formed in the same sedimentary and diagenetic environment are similar and consistent. Predicting the rock types in non-cored wells and non-cored sections in cored wells by using discriminant model has a certain degree of scientific and rationality. The quantitative discriminant method includes pre-processing of logging curves, standardization, selection of sensitive parameters, establishment of quantitative models, and comparative analysis of predictions.

### Pre-processing of logging curves

The pre-processing of logging data includes digitization of analog curves, depth correction, environmental impact correction and standardization^36–40^. In well logging operations, due to a series of reasons, such as irregular borehole, different types, shapes and weights of various downhole instruments, wellhead zeroing, in addition to improper correction of bottomhole friction force, the depth deviation of each logging curve in the same well occurs^41,42^. Natural gamma ray, compensated neutron, resistivity and compensated density logging are affected by random factors, resulting in abnormal points which are not consistent with formation information. Therefore, it is necessary to pre-process the original logging data before quantitative analysis to remove the abnormal information unrelated to the actual geological conditions, so as to avoid the deviation of rock type recognition, impacting the recognition results.

For the curve with abnormal points, a standard well is often selected, and the corresponding abnormal point is corrected to the value corresponding to the standard well. Correcting as the following formula:

1 $${\text{S}}_{{{\text{nom}}}} = {\text{L}}_{\min } + \left( {{\text{L}}_{\max } - {\text{L}}_{\min } } \right) \times \left( {{\text{S}}_{\log } - {\text{S}}_{\min } } \right){/}\left( {{\text{S}}_{\max } - {\text{S}}_{\min } } \right)$$

${\text{L}}_{{{\text{min}}}}$ and ${\text{L}}_{{{\text{max}}}}$ are the minimum and maximum values of logging curves in standard wells. ${\text{S}}_{{{\text{min}}}}$ and ${\text{S}}_{{{\text{max}}}}$ are the minimum and maximum values of logging curves in the well to be calibrated. ${\text{S}}_{{{\text{log}}}}$ is the logging value of the point to be calibrated, and ${\text{S}}_{{{\text{nom}}}}$ is the log value after calibration.

### Standardization of logging curves

Standardizing the logging curves including sonic time difference, natural gamma ray, spontaneous potential, and resistivity, so as to eliminate the systematic errors caused by drilling fluid, equipment, construction time and other factors between each well, making the overall characteristics of all curves in the target area conform to the actual geological characteristics. Presently the standardization is divided into the following steps:

1. Selecting the standard layer. Taking the stratum with wide distribution and stable velocity characteristics in the target area as the standard stratum. The selected standard layer must meet three conditions: ① drilled through by all wells in the area; ② having certain thickness and uniform distribution; ③ the nearest to the target layer.
2. Determining the standard value of the curve. Performing histogram statistics on some curve of the standard layer of all wells to find the standard value of the corresponding curve by using statistical analysis methods.
3. Determining the correction value of single curve in each well. Firstly, calibrating single well, and then calibrating other required wells in the same way.

### Selection of sensitive parameters

Each kind of rock has multiple petrophysical characteristics, such as radioactivity, porosity, and conductivity. There is a relative advantage relationship between these characteristics, that is, one or two advantages are prominent and dominant. For example, grainstone has good porosity, so high porosity is its dominant petrophysical characteristic. Correspondingly, its logging response features are characterized as low density, high neutron, low natural gamma, and high resistivity. Mudstone and wackstone have poor porosity and high mud content, so high radioactivity is their dominant petrophysical characteristics, resulting in high natural gamma ray and low resistivity in the logging response characteristics. The total porosity of packstone is large, but the logging response is characterized by low density, high neutron and medium resistivity resulted by the mud filled reducing the connectivity between the pores. The results show that the density and neutron logging can better reflect the lithology and physical properties.

**Figure 5:**
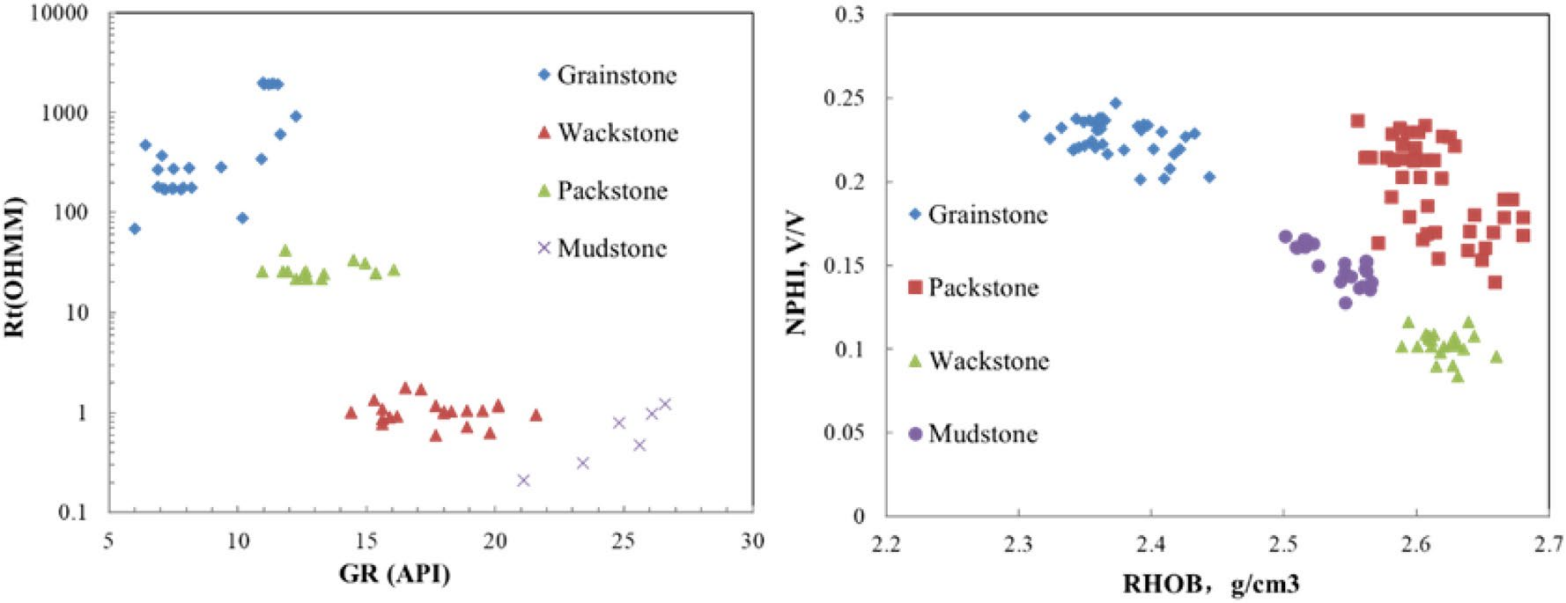
Cross plot of resistivity against natural gamma, neutron against density.

It can be seen from the cross-plot that the natural gamma-resistivity and neutron-density cross-plot (Fig. 5) can well distinguish grainstone, packstone, wackstone and mudstone. Therefore, the natural gamma, resistivity, neutron and density logging curves were selected as sensitive curves based on six wells for building discriminant model.

### Parameter normalization

On the basis of curve standardization, the parameter normalization method is used to acquire dimensionless density and neutron curves, and the range of the neutron and density curves is normalized to the interval [0,1] to ensure the discriminant model not affected by the dimension. The normalization formula is as follows:

2 $${\text{S}}_{{{\text{N}},{\text{i}}}} = \frac{{{\text{C}}_{{\text{i}}} - {\text{C}}_{\min } }}{{{\text{C}}_{\max } - {\text{C}}_{\min } }}\quad {\text{i}} = 1,2,3 \ldots ,{\text{n}}$$

where S_N,i_ is the value of various points of each normalized curve; Ci is the value of various points of the original curve; C_min_ is the minimum value among all sample points of the curve; C_max_ is the maximum value of all sample points of the curve; N is the number of sample points of the curve.

### Discriminant model of rock type

#### Basic principle of Bayesian discrimination

Given k populations G_1_, G_2_,…G_k_, whose m dimensional distribution density function are respectively $f_{1} \left( {\mathbf{X}} \right),f_{2} \left( {\mathbf{X}} \right), \ldots f_{k} \left( {\mathbf{X}} \right)$,the prior probability of each population is respectively $q_{1}$, $q_{2} \ldots$,$q_{k}$.For the sample ${\varvec{X}} = \left( {x_{1} ,x_{2} \ldots ,x_{m} } \right)^{T}$,it is needed to determine which population to be attributed to. Regarding **X** as a point in the Euclidean space with m dimensions, the Bayesian criterion expects to achieve a division of the sample space:$R_{1} ,R_{2} \ldots ,R_{k}$.In this way, a discriminant rule is built, i.e., if **X** falls into $R_{i} \left( {i = 1,2 \ldots ,k} \right)$,then **X**$\in G_{i}$, where $R_{i} = \left\{ {{\varvec{X}}:y_{i} \left( {\varvec{X}} \right) = \max_{j} y_{j} \left( {\varvec{X}} \right)} \right\}$, $y_{i} \left( {\varvec{X}} \right) = q_{i} f_{i} \left( {\mathbf{X}} \right),\;\left( {i,j = 1,2, \ldots ,k} \right)$.

When $$G_{i} \sim N_{m} \left( {\mu_{i} ,\Sigma_{i} } \right)$$,then

3 $$y_{i} \left( {\varvec{X}} \right) = q_{i} \left( {2\pi } \right)^{{ - \frac{m}{2}}} \left| {{\Sigma }_{i} } \right|^{{ - \frac{1}{2}}} {\text{exp}}\left\{ { - \frac{1}{2}\left( {{\varvec{X}} - {\varvec{\mu}}_{{\varvec{i}}} } \right)^{T} {\Sigma }_{i}^{ - 1} \left( {{\varvec{X}} - {\varvec{\mu}}_{{\varvec{i}}} } \right)} \right\}$$

After taking the logarithm, removing irrelevant terms, this Eq. (3) is simplified as:

4 $$Z_{i} \left( {\varvec{X}} \right) = \ln q_{i} - \frac{1}{2}\left( {\ln \left| {{\Sigma }_{i} } \right| + {\varvec{X}}^{T} {\Sigma }_{i}^{ - 1} {\varvec{X}} + {\varvec{\mu}}_{{\varvec{i}}}^{{\varvec{T}}} {\Sigma }_{i}^{ - 1} {\varvec{\mu}}_{{\varvec{i}}} } \right) + {\varvec{X}}^{T} {\Sigma }_{i}^{ - 1} {\varvec{\mu}}_{{\varvec{i}}}$$

where $$i = 1,2, \cdots ,k,$$ if

5 $$Z_{i} \left( {\varvec{X}} \right) = \mathop {\max }\limits_{j} y_{j} \left( {\varvec{X}} \right)$$

then **X**$$\in G_{i}$$.

Equation (4) is the quadratic function with the covariance matrix of k populations. Its actual calculation load is very large. Thus, it is further assumed that the covariance matrix of the population is the same, i.e.,$\Sigma = \Sigma_{1} = \Sigma_{2} = \cdots = \Sigma_{k}$.When the parameters of population are unknown, they could be estimated by typical samples of population. Given the size of typical sample of $G_{i}$ is $n_{i}$, mean value is $\overline{X}^{\left( i \right)}$, deviation matrix is $L_{xx}^{\left( i \right)}$, $i = 1,2, \ldots ,k$, $\mathop \sum \limits_{i = 1}^{k} n_{i} = n$, $\mathop \sum \limits_{i = 1}^{k} L_{xx}^{\left( i \right)} = L_{xx}$, $S = {\hat{\Sigma }} = \frac{1}{n - k}L_{xx}$, then discriminant function could be simplified as

6 $$\begin{aligned} Z_{i} \left( {\varvec{X}} \right) & = \ln q_{i} - \frac{1}{2}\left( {\overline{\user2{X}}^{\left( i \right)} } \right)^{T} S^{ - 1} \overline{\user2{X}}^{\left( i \right)} + {\varvec{X}}^{T} S^{ - 1} \overline{\user2{X}}^{\left( i \right)} \\ & = \ln q_{i} + c_{0i} + c_{1i} x_{1} + c_{2i} x_{2} + \cdots + c_{mi} x_{m} \\ & = \ln q_{i} + c_{0i} + C_{i}^{T} \overline{\user2{X}} \\ \end{aligned}$$

where $q_{i} = \frac{{n_{i} }}{n}$, $C_{i} = \left( {c_{1i} ,c_{2i} , \ldots ,c_{mi} } \right)^{T} = S^{ - 1} \overline{\user2{X}}^{\left( i \right)} , \;c_{0i} = - \frac{1}{2}\left( {\overline{\user2{X}}^{\left( i \right)} } \right)^{T} S^{ - 1} \overline{\user2{X}}^{\left( i \right)}$. Discriminant rule is still Eq. (5).

If $Z_{m} \left( {\varvec{X}} \right) = \max_{1 \le i \le k} \left\{ {Z_{i} \left( {\varvec{X}} \right)} \right\}$, then **X** is attributed as *m*th class. The posterior probability $P\left( {i\left| {\varvec{X}} \right.} \right)$ of **X** attributed to the *i*th class is:

7 $$P\left( {i\left| {\varvec{X}} \right.} \right) = \frac{{{\text{exp}}\left[ {Z_{i} \left( {\varvec{X}} \right) - Z_{m} \left( {\varvec{X}} \right)} \right]}}{{\mathop \sum \nolimits_{j = 1}^{k} {\text{exp}}\left[ {Z_{j} \left( {\varvec{X}} \right) - Z_{m} \left( {\varvec{X}} \right)} \right]}}$$

#### Bayesian discriminant model

The Bayesian stepwise discriminant method is applied to establish the recognition model of various rock types based on the selected sensitive logging parameters. The credibility Cm* is used to test the significance of the difference between the whole set of the established discriminant model and each rock type, so as to ensure obvious effect of the recognition model.

Different rock types have different discriminant function values Fm (m = 1,2,3,4). For the new rock sample to be judged, following the principle of making the discriminant function reach the maximum value, then classifying it as the m-th rock type:

8 $${\text{Fm}}^{*}\left( {\text{S}} \right) = \mathop {\max }\limits_{{1 \le {\text{m}} \le 4}} \left\{ {{\text{F}}_{{\text{m}}} \left( {\text{S}} \right)} \right\}$$

where Fm(S) is the function value obtained by substituting the corresponding sensitive logging parameters of the rock sample to be judged into the discriminant formula of each rock type. Fm*(S) is the maximum value of the discriminant function values of all rock types for the rock sample to be judged. M is the number of rock types, ranging from 1 to 4. m* is the rock type with the largest discriminant function value. S is the normalized parameter value of the input sensitive logging parameters. S1 expressed as NPHI.S2 expressed as RHOB.S3 expressed as GR. S4 expressed as Rt.

The discriminant model for rock types based Bayesian theory and logging data in the study area is as follows:

9 $${\text{F1}} = {377}.{\text{858S1}} + {219}.{\text{375S2}} - {4}.{\text{912S3}} + {1}.{\text{882S4}} - {31}0.{156}$$

10 $${\text{F2}} = {365}.{\text{462S1}} + {217}.{\text{632S2}} - {3}.{\text{949S3}} + {1}.{\text{462S4}} - {3}00.{689}$$

11 $${\text{F3}} = {359}.0{\text{28S1}} + {216}.{\text{236S2}} - {3}.{\text{162S3}} + {1}.{\text{218S4}} - {298}.{328}$$

12 $${\text{F4}} = {336}.{\text{252S1}} + {2}0{8}.{\text{484S2}} - {2}.{\text{936S3}} + {1}.0{\text{49S4}} - {282}.{461}$$

F1 expressed as grainstone. F2 expressed as packstone. F3 expressed as wackstone. F4 expressed as mudstone.

The credibility that the rock sample to be judged belongs to the m* rock type is:

13 $${\text{Cm}}^{*} = \frac{{{\text{exp}}\left[ {{\text{F}}_{{{\text{m}}^{*} }} \left( {\text{V}} \right)} \right]}}{{\mathop \sum \nolimits_{{{\text{m}} = 1}}^{4} {\text{exp}}\left[ {{\text{F}}_{{\text{m}}} \left( {\text{V}} \right)} \right]}}$$

where Cm* is the credibility value of the rock sample to be judged belonging to the m* rock type. Fm(V) is the function value of the logging parameters of the rock sample to be judged substituted into each logging discriminant formula. Fm* (V) is the maximum value of all discriminant function values of the rock sample to be judged. m is the number of rock types, ranging from 1 to 4, and m* is the rock type with the largest discriminant function value.

#### Verification of discriminant model of rock type

In present study, examining of the original sample and external reliability verification are used to test the established discriminant model of rock type. In the original sample back-judgment mode, the samples participating in training were tested by themselves, while the external reliability verification was conducted by the cored wells not participating in training.

##### Examining of original sample

**Figure 6:**
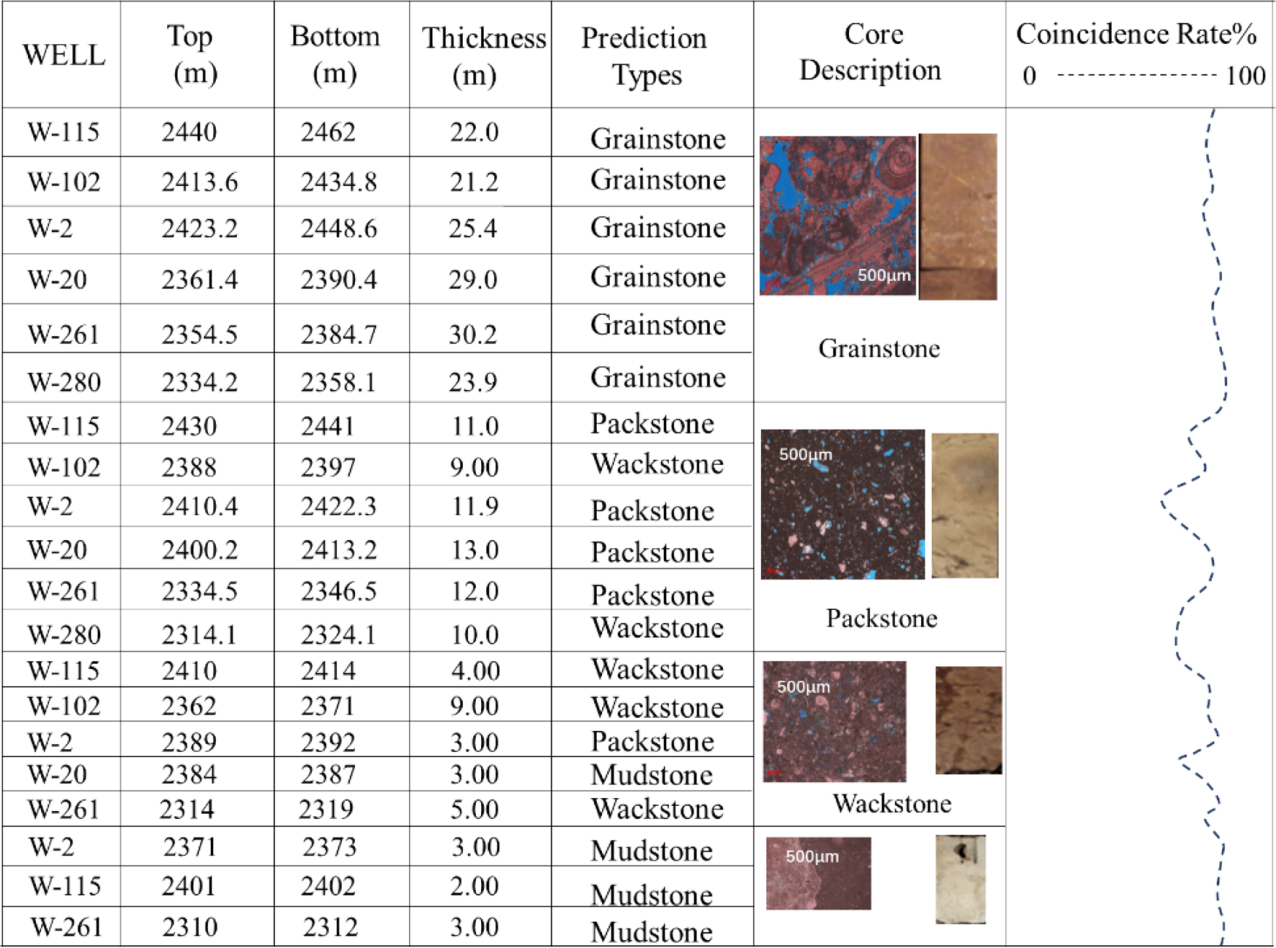
Test results of discriminant model for examined samples.

The verification was performed on the 144 samples participating in the training. The results are shown in the figure and table below (Fig. 6, Table 2). The established Bayesian discriminant model has an accuracy rate of 83.65% for the original sample, and the cross-confirmation accuracy for the original sample is 81.98%. These two accuracies are very close, indicating that the established rock type discriminant model is relatively stable. It can be seen from Table 2 that the accuracy of examining and cross-confirmation of grainstone and mudstone are relatively high, while that of packstone and wackstone are relatively low.

**Table 2:** Examining results of training samples.

	Rock types	Grainstone	Packstone	Wackstone	Mudstone	Total
**Back-judgment results of the original samples**						
Number	Grainstone	39	1	0	0	40
	Packstone	0	32	8	0	40
	Wackstone	0	3	27	6	36
	Mudstone	0	0	5	23	28
Percentage (%)	Grainstone	97.5	2.5	0.0	0.0	100
	Packstone	0.0	80.0	20.0	0.0	100
	Wackstone	0.0	8.3	75.0	16.7	100
	Mudstone	0.0	0.0	7.9	82.1	100
**Cross-confirmation**						
Number	Grainstone	38	2	0	0	40
	Packstone	3	30	7	0	40
	Wackstone	0	4	26	6	36
	Mudstone	0	0	4	24	28
Percentage (%)	Grainstone	95.0	5.0	0.0	0.0	100
	Packstone	7.5	75.0	17.5	0.0	100
	Wackstone	0.0	11.1	72.2	16.7	100
	Mudstone	0.0	0.0	14.3	85.7	100

**Figure 7:**
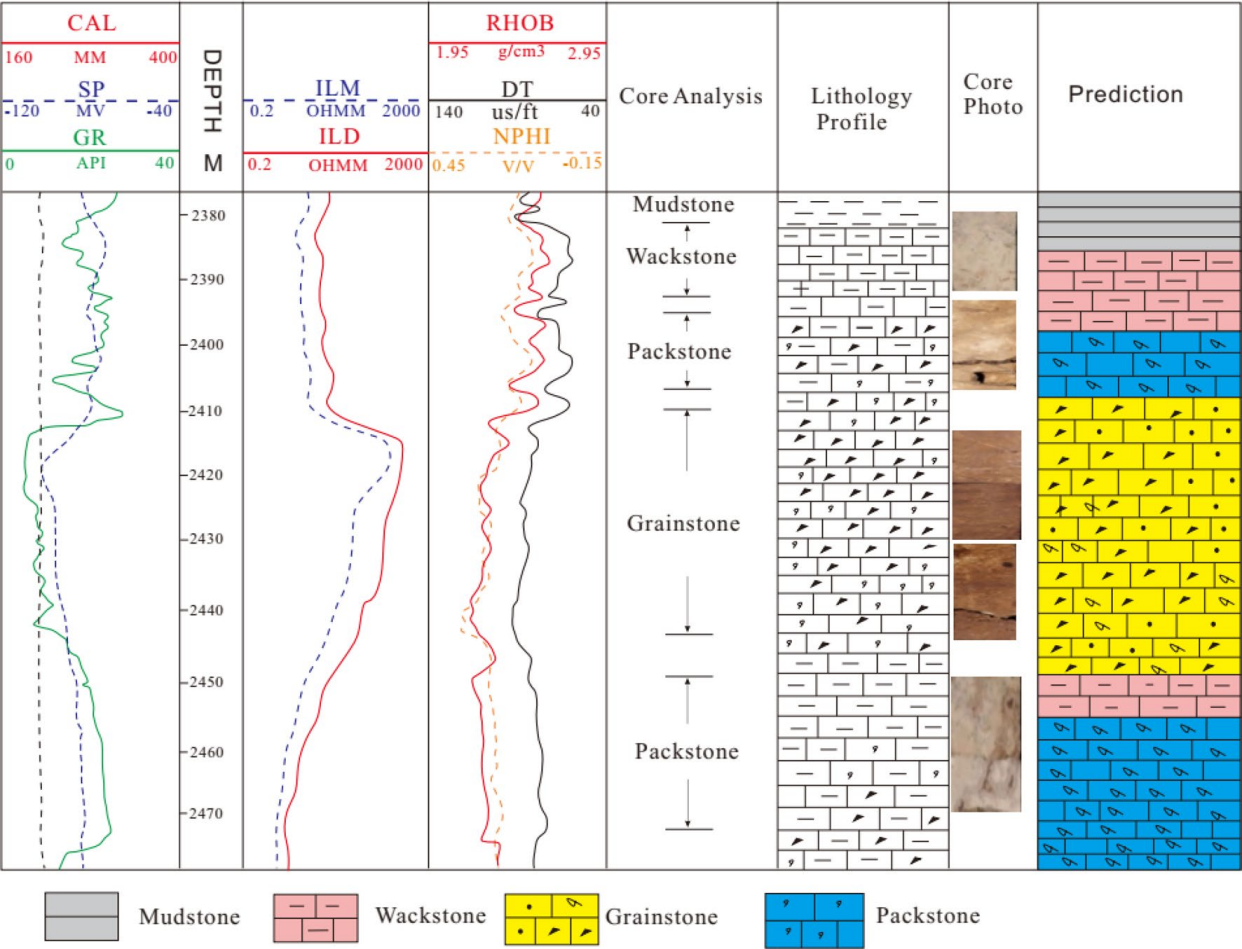
Predicting results of well W-11 based on Bayesian discriminant model.

It can be seen from Fig. 7 that there is a possibility that packstone misjudged as wackstone, and wackstone misjudged as mudstone (Fig. 7).

**Figure 8:**
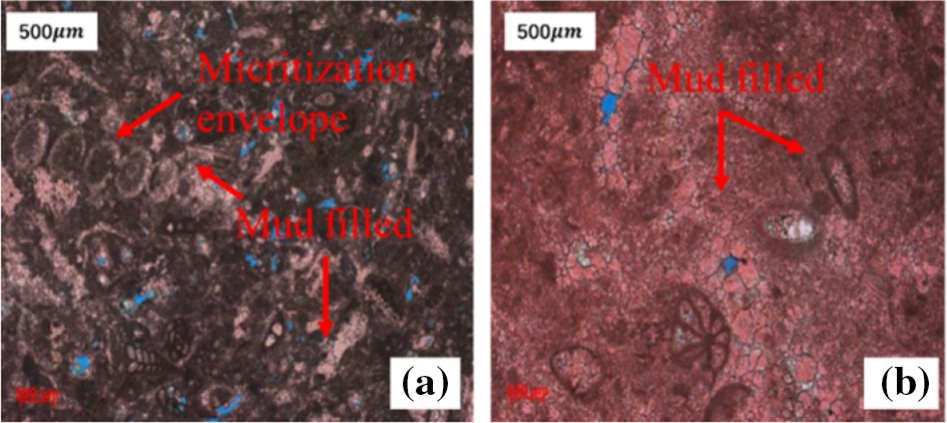
Comparison of thin sections of packstone (**a**) and wackstone (**b**) at 2450–2451 m in Well W-11.

The reasons for the misjudgment are as follows: (1) Similar sedimentary facies. The sedimentary environment of grainstone and mudstone, determines that their rock fabrics have obvious differences compared with other types of rocks. These two types of rocks can only be generated in one unique sedimentary environment. Grainstone is mainly developed in the high-energy environment of platform marginal reefs, while mudstone is mainly developed in the closed environment of restricted platform. In contrast, wackstone and packstone can be developed in the transition area of two sedimentary facies belts. Because packstone is generally developed in the sedimentary facies of the platform front slope, where the hydrodynamic force is weak, resulting in poor pore structure, and logging response characteristics of the density, neutron, and resistivity similar to wackstone developed on the confined platform, which is easy to cause misjudgment. (2) Thin formation thickness. For wackstone and mudstone with poor pore structure and high mud content, the thin layer will also lead to similar logging response characteristics, resulting in the misjudgment of wackstone marl as mudstone. (3) Micritization. After micritization, the rock fabric and pore structure of packstone are difficult to distinguish from the wackstone because of mud filled (Fig. 8). (4) The small number of samples. For the stratum with few cores, because of the limited number of samples, it is easy to make misjudgments. The more samples involved in training, the higher the accuracy of prediction.

##### External reliability verification

The established discriminant models of rock types were applied to the other cored wells in the study area that were not participated in the training, such as well W-11, to verify the accuracy of the above method. The cored interval of well W-11 is 2360–2480 m, the total core footage is 120 m, and the total length of cored core is 112 m, as well as the average harvest rate is 97.5%. The logging data of this well is corrected for depth and wellbore environment impact. Compared with the description of logging and core, it can be seen that the Bayesian discriminant model has a good application effect in predicting rock type, and the rock type recognized conformed to the description of logging with the coincidence rate 90%. According to the predicted results, it can be seen that the discriminant accuracy of grainstone and mudstone almost approached 100%. Around 2390–2400 m and 2450–2459 m, mudstone is misjudged as wackstone, while between 2385 and 2390 m, packstone is wrongly judged as wackstone (Fig. 7).

According to predicting results of well W-11 (Fig. 7), it can be seen that the conclusions of the external verification are consistent with the ones of the re-judgment. The discriminant accuracy of grainstone and mudstone is the highest, as high as 90%. Misjudgments have been made for both packstone and wackstone. The main reason for errors in external reliability verification is mainly related to the sedimentary environment and diagenesis, which make the pore structure of grainstone and mudstone have no similarity with other rock types, resulting in unique and specific logging response characteristics. However, due to the similarity of sedimentary environments and the impact of compaction, similarity of the pore structure and rock fabric exist in some wackstone and packstone, leading to ambiguity of the logging characteristics of the rock, which ultimately affects the discriminant accuracy.

According to the predicting results of well W-11 (Fig. 7), it can be seen that though existing misjudgment, the overall conformity is still over 80%, which meets the actual production requirements. Therefore, this method can be popularized and applied to predict rock types in the Middle East region where there is no cored well or no cored interval in the cored well.

### Local distribution of rock types

**Figure 9:**
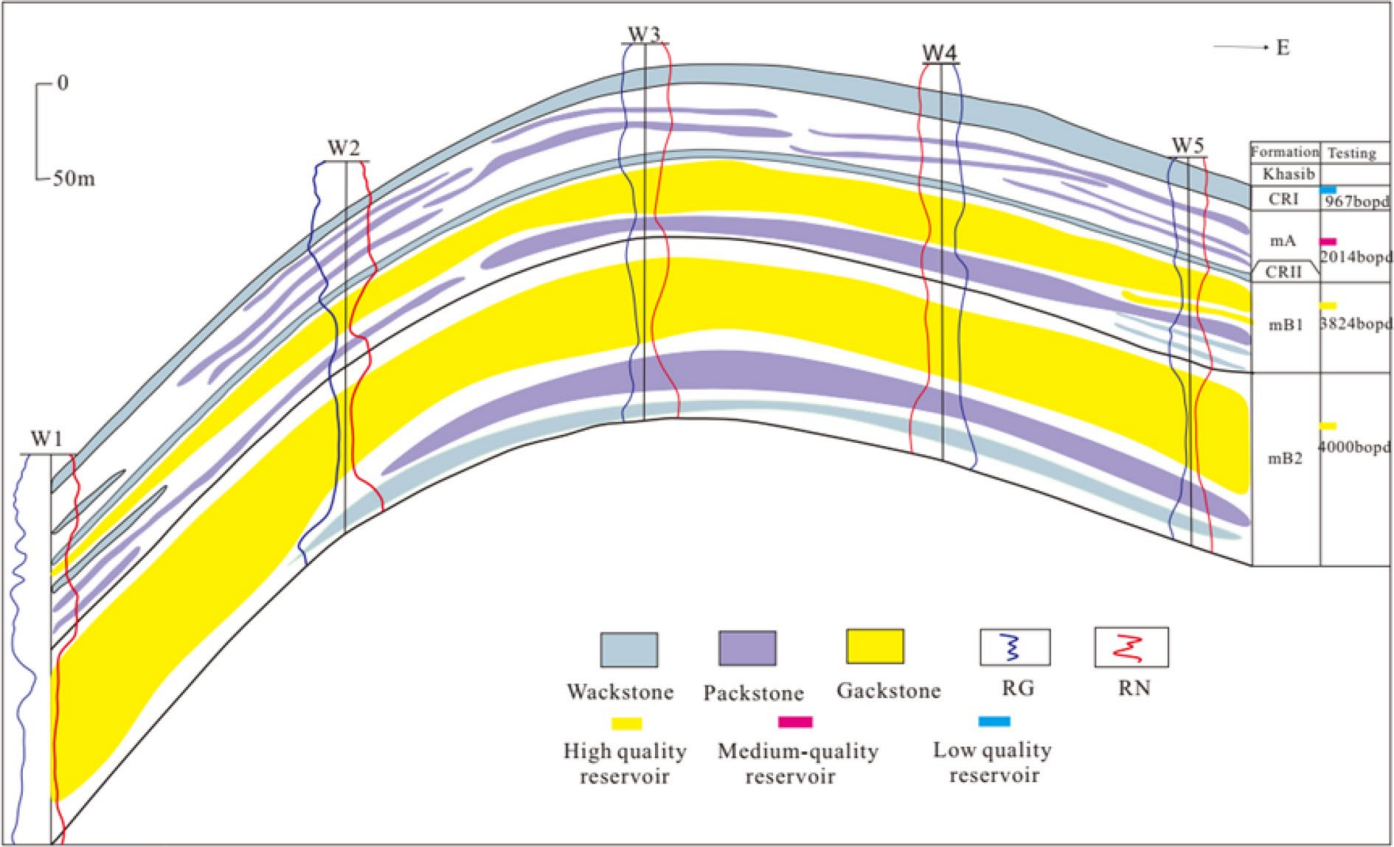
East–west section of rock type distribution in the study area.

The Bayesian discriminant model is used to predict the rock types in the study area, achieving the rock type distribution profile in the study area (Fig. 9). According to the distribution profile, it can be seen that continuous distribution of grainstone and wackstone are widely developed in the study area. Among them, the grainstone is distributed in the upper part of mB1 and mB2, with a large thickness and strong continuity, where produces 3824 bopd daily. These two layers are obviously high-porosity, high-permeability and high-quality reservoirs. The wackstone is mainly distributed in the CRI and CRII sections, with strong continuity but thin thickness. The oil test shows that the daily oil production at these two sections is only 967 bopd, which is a low-quality reservoir. The packstone is scattered and randomly distributed, unevenly distributed, and not continuous. The oil test shows that the daily oil production at this type of reservoir is 2014 bopd, which is a medium-quality reservoir. From the perspective of sedimentary facies, the mB2 member is located in a platform marginal reef environment, with high water energy, and the thickness of the anticline axis is better than that of the two wings, developing grainstone well. The upper part of mB1 is located in the transition zone between open platform and platform marginal reef, where the water body has medium energy and grainstone is also developed. CRI and CRII are confined platforms with weak water energy and mainly developed mud. The mA section is affected by the restricted platform and the water energy is not high, which leads to the unstable sedimentation of the area, and the developed packstone is discontinuously distributed. Therefore, the distribution of rock types predicted in the study area matches the actual sedimentary environment in the area, which confirms the validity, applicability and generalizability of the Bayesian discriminant model.

## Discussions

Taking W Oilfield as an example, the discriminant model of rock type established in this paper has a very high accuracy and overall consistency. However, two main factors, sedimentary environment and diagenesis, may affect the discrimination effect. They may lead to the convergence of logging response characteristics and affect the discrimination accuracy of the model, which can be further improved by increasing the number of rock samples subsequently. In terms of calculation method, the covariance inverse matrix data ${ }A_{ij}^{ - 1}$ of the rock samples that participated in the training may affect the independence of the discriminant model in the model calculation, and the smaller value of the normalized logging data will result in the larger value of the covariance inverse matrix data $A_{ij}^{ - 1}$ in the calculation process of the discriminant model of rock type, which will produce some systematic errors. Drawn from the prediction results, it can be seen that the Bayesian stepwise discriminant method still has some shortcomings, but compared with the results of core analysis, the overall discriminant results show that this method can still be well adapted to the quantitative logging recognition of rock types in the study area. At the same time, the Bayesian discriminant model makes up for the shortcomings of the method based on capillary pressure curve, NMR logging and fluid unit, and solves the problem of rock type recognition in the carbonate reservoir in Iraq. For porous carbonate reservoir, the calculation result of Bayesian discriminant model is reasonable and the method is appropriate.

## Conclusions

1. The Bayesian discriminant model is established based on the logging parameters of natural gamma ray, resistivity, neutron, and density, which are sensitive to rock types, which integrates multiple logging parameter information and conforms to the actual geological characteristics.
2. The accuracy of the Bayesian discriminant model on the original sample and cross confirmation have reached more than 80%. The accuracy of predicting grainstone and mudstone has reached more than 90%. Affected by factors such as depositional environment and compaction, the rock fabric and pore structure of packstone and wackstone, wackstone and mudstone are not significantly different, which makes the corresponding logging response characteristics appear more decomposability, resulting in misjudgment in the process of recognizing packstone and wackstone.
3. Although the Bayesian discriminant model has misjudgments on the packstone and mudstone, the overall accuracy is still as high as 80% or more. The distribution of the predicted rock types in the region can match the actual sedimentation, which can meet the needs of the production and development in the Middle East oil fields.
