# Author Correction: Study on discriminant method of rock type for porous carbonate reservoirs based on Bayesian theory

**DOI:** 10.1038/s41598-021-99325-6

**Published:** 2021-09-28

**Authors:** Xinxin Fang, Hong Feng

**Affiliations:** 1 China Coal Research Institute, Beijing, 100013 China; 2 China Coal Technology and Engineering Group Xian Institute, Xi’an, 710077 Shaanxi China

Correction to: *Scientific Reports* 10.1038/s41598-021-98154-x, published online 20 September 2021

In the original version of this Article Xinxin Fang and Hong Feng were incorrectly listed as equally contributing authors. Consequently, the equal contribution statement has now been removed.

The original Article has been corrected.

